# A seeding-searching-ensemble method for gland segmentation in H&E-stained images

**DOI:** 10.1186/s12911-016-0312-5

**Published:** 2016-07-21

**Authors:** Yizhe Zhang, Lin Yang, John D. MacKenzie, Rageshree Ramachandran, Danny Z. Chen

**Affiliations:** 1 Department of Computer Science and Engineering, University of Notre Dame, IN, 46556 USA; 2 Department of Radiology & Biomedical Imaging, University of California, San Francisco, 94143 CA USA; 3 Department of Pathology, University of California, San Francisco, 94143 CA USA

## Abstract

**Background:**

Glands are vital structures found throughout the human body and their structure and function are affected by many diseases. The ability to segment and detect glands among other types of tissues is important for the study of normal and disease processes and helps their analysis and visualization by pathologists in microscopic detail.

**Methods:**

In this paper, we develop a new approach for segmenting and detecting intestinal glands in H&E-stained histology images, which utilizes a set of advanced image processing techniques: graph search, ensemble, feature extraction, and classification. Our method is computationally fast, preserves gland boundaries robustly and detects glands accurately.

**Results:**

We tested the performance of our gland detection and segmentation method by analyzing a dataset of over 1700 glands in digitized high resolution clinical histology images obtained from normal and diseased human intestines. The experimental results show that our method outperforms considerably the state-of-the-art methods for gland segmentation and detection.

**Conclusions:**

Our method can produce high-quality segmentation and detection of non-overlapped glands that obey the natural property of glands in histology tissue images. With accurately detected and segmented glands, quantitative measurement and analysis can be developed for further studies of glands and computer-aided diagnosis.

## Background

**Fig. 1:**
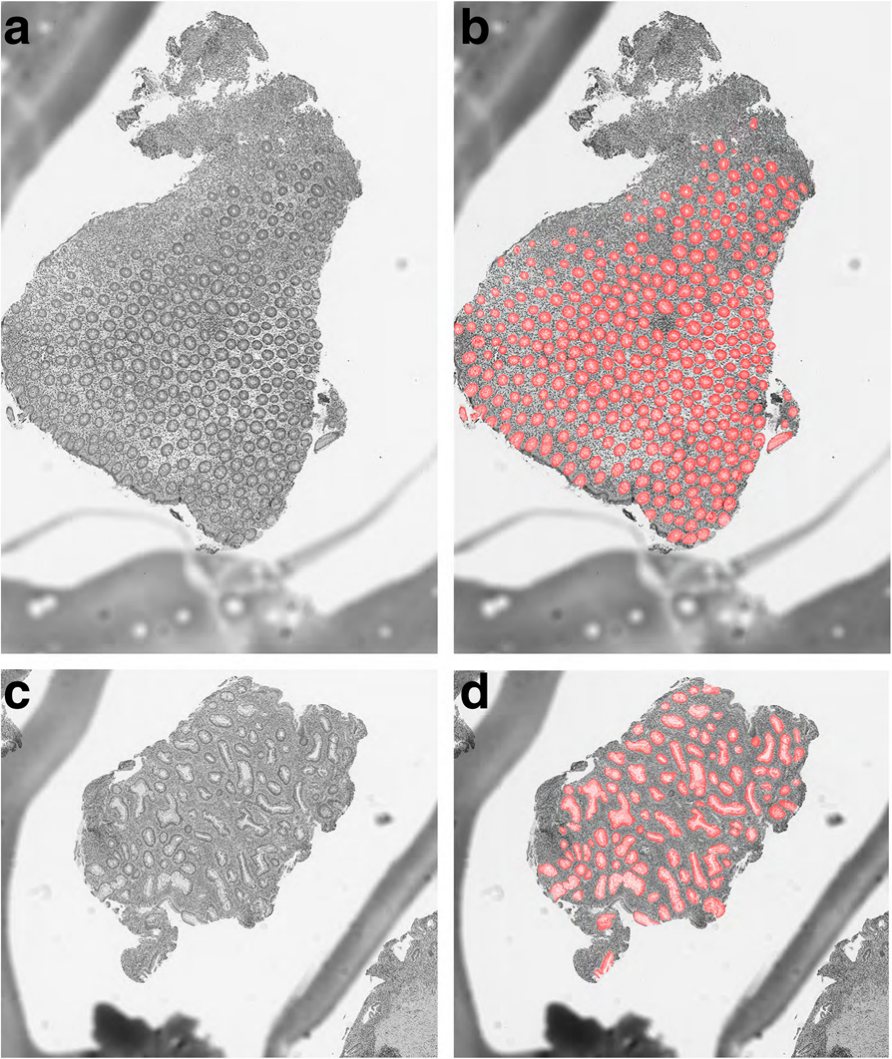
Example of gland segmentation in normal and diseased tissue. *Left*: High resolution histology images of normal glands (**a**) and glands with architectural distortion from chronic inflammation (**c**). *Right*: Segmentation output of the normal (**b**) and chronically inflamed tissue (**d**) with the glands highlighted in red

Glands are well-organized structures found throughout the body and are primarily responsible for the storage and secretion of bodily fluids. Glands in the gastrointestinal tract secrete materials which help lubricate and protect the tissues that line the inner surface of the gut. Alterations in gland structures are used extensively to help medical diagnosis. Examples include architectural distortions produced by inflammatory bowel disease [15] and the clustering of glands in prostate cancer [16]. In order to quantitatively analyze the appearance of glands with computer-aided techniques, an important step is to accurately segment and detect individual glands [10, 11] (Fig. 1). Hematoxylin and eosin stained tissue specimens are often used as the “gold standard” for histological analysis, and are routinely used for clinical diagnosis and treatment monitoring. Several major challenges for gland detection on H&E-stained histology images include the complex image background (e.g., multiple cell types and tissue regions), variations in gland morphology, weak gland boundaries caused by variations in sectioning, variations in staining, variable presentation of disease, etc.

**Fig. 2:**
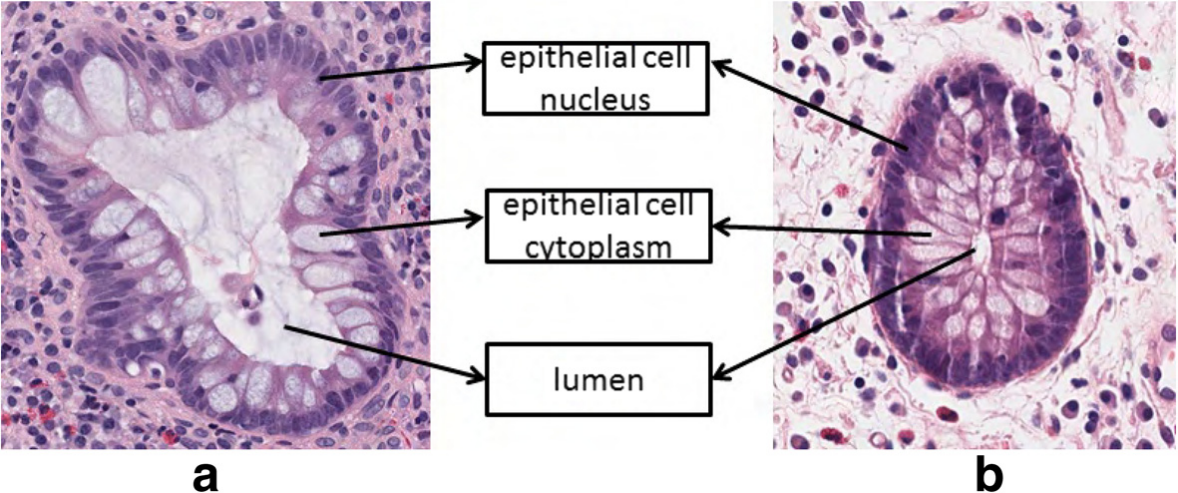
The basic components of in diseased (**a**) and normal (**b**) glands

A gland is composed of multiple epithelial cells organized in an oval or circle around a lumen, see Fig. 2. A common known gland detection approach was based on identifying the basic objects in tissue images (e.g., lumen and cell nucleus), and colors have been intensively explored to help detect such components. In [11], a tissue image was first decomposed into a set of primitive objects, e.g., nucleus, lumen, stroma (stroma is the material between glands), and then glands were segmented by utilizing the organizational information of such objects. A similar idea was incorporated in [16], which first detected possible lumen areas, and then identified gland borders by an expansion algorithm. A major improvement was attained in [9, 10], which detected glands by directly finding the gland epithelial cell nucleus borders; a conditional random field model with a cost-based soft contour smoothness constraint was proposed to find gland epithelial cell nucleus borders in the polar space, the enclosed region of a contour thus obtained was viewed as a segmentation proposal, and its type was determined by a typical feature extraction and classification process. Recently, a deep learning method was utilized to simultaneously detect glands and specialized small intestinal structures called villi in H&E-stained images [19]; similar to [9], gland region proposals were first generated based on domain knowledge, and then a convolutional neural network (CNN) [13] was trained and applied to determine the region types. Although the method in [19] achieved better performance than previous methods, deep learning models are generally quite time-consuming to train and apply. To reduce the time cost to acceptable level for clinical practice, it requires high-end hardware to support the intensive computation [13].

**Fig. 3:**
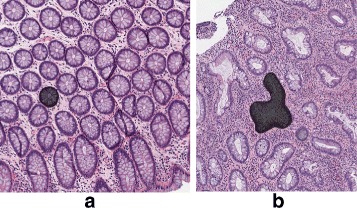
The diversity of gland shapes and the submucosal cells surrounding glands in normal colon (**a**) and colon with architectural distortion in a patient with chronic colon inflammation (**b**). One gland in each image is marked in black

The rich and diverse appearance of gland shapes and their cellular background (submucosa) present challenges for the the design of robust computer-aided gland detection strategies. As one may see in Fig. 3, glands can be densely neighbored, background regions and gland regions can look similar, parts of gland borders can be quite unclear, and due to various inflammations or diseases, glands can appear in irregular shapes. These issues can cause considerable difficulties to preserving correct gland boundaries and preventing segmentation leaking. Segmentation leaks occur when the area or volume of segmentation expands beyond the actual target structure to include other glands and/or surrounding submucosal cells. Leaking in segmentation can downgrade the detection performance and cause further potential problems to quantitative analysis (e.g., analyzing the shapes and morphology of glands). The previous gland detection and segmentation methods [9–11, 16, 19] were not designed for preserving the true gland boundaries and preventing segmentation leaking. Consequently, their performance can be downgraded in noisy and complicated imaging scenarios (e.g., in H&E-stained images).

In this paper, we develop a robust gland segmentation and detection approach, which is capable of preserving true gland boundaries and reducing segmentation leaking. First, we design a stable and effective ensemble procedure based on graph search to generate good quality boundary-preserving gland segmentation proposals. Then, we apply a feature extraction and classification procedure to the generated proposals to determine their types(gland or non-gland). To further eliminate potential false positives, we propose a process to re-weight the gland probability for each segmentation proposal produced by the classification (regression) procedure. Finally, a region merging step is designed for handling some over-segmentation cases with irregular-shaped glands. Our method improves the gland segmentation and detection performance by producing better boundary-preserving and less leaked segmentation proposals. Comparing to [9, 10], we are able to identify more accurate gland borders. In general, we achieve several technical improvements. (1) From the segmentation method point of view, we utilize a graph search procedure to determine the gland boundaries. This procedure allows us to impose a hard and more stable geometric smoothness constraint on the boundary searching. The smoothness constraint is natural for glands, and sometimes is even critical for preventing leaking. Rather than using penalty terms in the cost functions to ensure boundary smoothness, the structural constraint in the graph search method is more stable and robust to noise. After searching, since not every boundary found is entirely correct, we cannot solely rely on each individual searched result. We generate a more reliable and robust gland boundary probability map (BPM) by collecting and collaborating these individual boundary results by an ensemble procedure.

(2) From the output of the segmentation method point of view, based on the generated boundary probability map, we produce non-overlapped segmentation proposals, rather than proposal regions with arbitrary overlapping. This is mainly because glands in tissue images naturally seldom overlap. We thus have much less regions proposed and these regions are mutually non-overlapped. This allows our method to utilize much faster and more advanced procedures.

(3) To further reduce false positives and over-segmentation cases, we design a local re-weighting step and a region merging step for post-processing.

In experiments, we show that our method can effectively preserve true gland boundaries and prevent segmentation leaking. Comparing to the state-of-the-art methods [9, 10], which also aimed to obtain good quality gland segmentation, we achieve better gland segmentation performance (by 6 % in recall and 8 % in precision), and consequently, better gland detection performance (by 6 % in recall and 6 % in precision).

## Method

### Overview

**Fig. 4:**
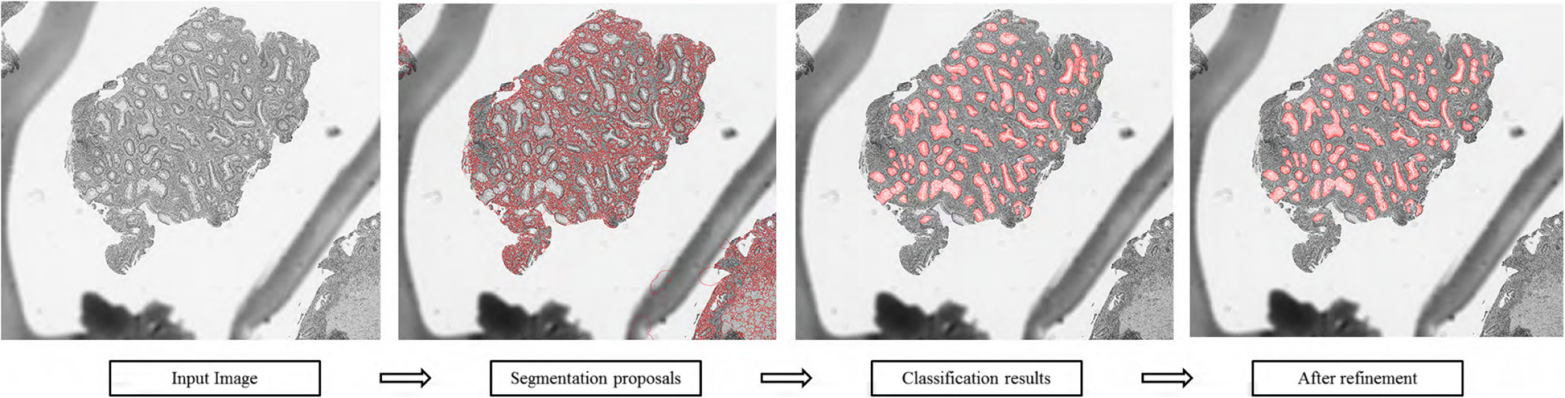
The overall processing flow of our method

In Fig. 4, we outline the overall processing flow of our method: We first generate segmentation proposals; then on each proposal region, we extract representative features; next, based on the extracted features, we classify the proposal regions as gland or non-gland by a handcrafted feature based classification procedure; finally, we apply a post-processing to further eliminate potential false positives and possible over-segmentation cases. The following sections show the details of our major steps one by one.

### Generating segmentation proposals

The contour of a gland epithelial nucleus border can be used to specify the occupation of a gland (e.g., see Fig. 2). After we obtain good gland boundaries, generating gland segmentation proposals from such boundaries is a relatively easy task. We first describe our gland boundary identification process. Then we show how to generate gland segmentation proposals from the identified gland boundaries.

#### Sampling and searching

**Fig. 5:**
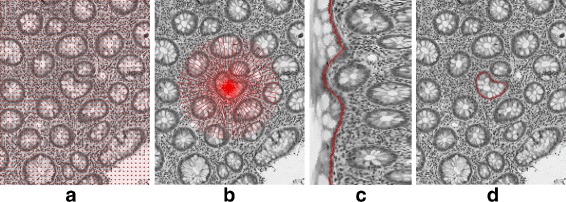
Finding an optimal closed contour by local search for every seeding point: (**a**) Seeding point sampling in an input image; (**b**)–(**d**) illustrating the search of a closed contour for one seeding point

The processes of sampling and searching aim to obtain independent gland boundaries for the subsequent ensemble step. We first choose a set of seeding positions on the input image, and then perform local search on each seeding point to find contours that correspond to gland epithelium nucleus borders. We seek to introduce as little bias as possible in the sampling, and thus simply apply a uniform grid in this sampling step (see Fig. 5a). We apply a relatively dense uniform grid for the need of covering small-sized and densely neighbored glands. To avoid unnecessary computation, we further prune away seeds with very low intensity (dark) by a conservative threshold.

For every seeding point, we want to find the closest contour of some epithelial cell nucleus border around it. For a seeding point inside a true gland, the gland boundary thus founded is the desired gland border contour covering that seed. For a seeding point outside all glands, there can still be portions of border contours from different glands around that seed, and the “contour” thus formed is supposed to cover these portions of borders without containing any gland body in its interior. Note that each seeding location, no matter it is inside or outside a gland, can both serve the purpose of helping generate gland boundaries which separate glands and non-gland tissue regions apart.

As a general rule-of-thumb, cell nuclei are stained darkly (with low image intensity) and colored deep purple. This is true for a variety of cell types including gland and epithelial cells as well as the immune and connective tissue cells in the submucosa surrounding the glands. Hence, during the search for a seeding point, we seek to find a continuous dark contour in the local image area containing that seeding point. We model the neighboring image area around a seeding point by a directed acyclic graph (DAG), and compute a cyclic shortest path in this graph which corresponds to the desired continuous dark contour in the image area (see Fig. 5). Below we discuss the details of this local search process.

**Fig. 6:**
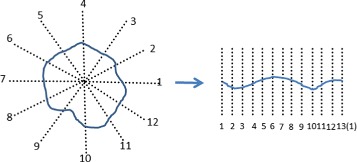
The polar transform and the corresponding multi-column graph *G*

From each seeding point *p*, we evenly shoot *n* rays, each of a length (or radius) *r*, around 360 degrees centered at *p*. On each ray, we evenly sample *m* positions, and construct a multi-column graph *G* that consists of *n* columns, such that each column corresponds to one ray and has *m* nodes (see Fig. 6). Note that the sampled positions on the *n* rays are in general different from the (grid) sampled seeding points.

We denote a node in *G* as *V*_*ij*_, which corresponds to the point (pixel) in the input image on the *j*-th ray (i.e., *j*-th column of *G*) and the *i*-th sampled position of that ray. We add a directed edge from *V*_*ij*_ to each $V_{i'j'}\phantom {\dot {i}\!}$ if *j*^′^=*j*+1 and |*i*−*i*^′^|≤1. Note that the constraint of |*i*−*i*^′^|≤1 is a geometric smoothness constraint which specifies that the sought contour must be sufficiently smooth with respect to its seeding point *p*. We define *w*_*ij*_, the node weight of *V*_*ij*_, as follows:

1 $$ w_{ij}=I(i,j)+\lambda\sum\limits_{i'=1}^{i} E(i',j)  $$

where

2 $$ {}I(i,j)=Intensity\left(x+\frac{ir}{m}cos\left(\frac{2\pi j}{n}\right),y+\frac{ir}{m}sin\left(\frac{2\pi j}{n}\right)\right)  $$

3 $$ E(i,j)=Edge\left(x+\frac{ir}{m}cos\left(\frac{2\pi j}{n}\right),y+\frac{ir}{m}sin\left(\frac{2\pi j}{n}\right)\right)  $$

and (*x*,*y*) is the position of the seeding point *p*. *Intensity*(*x*^′^,*y*^′^) is the intensity of pixel (*x*^′^,*y*^′^) in the original image. *Edge*=*Sobel*(*Intensity*), where *Sobel*(·) is the Sobel filter [17]. The term $\lambda \sum \limits _{i'=1}^{i} E(i',j)$ for *w*_*ij*_ in Eq. (1) is a region-based cost which discourages the sought path to cover too many edges resulted from the Sobel filter. It simply serves the purpose of preventing systematic leaking from closely neighbored glands. The parameter *λ* in this term is used to control the importance of the region cost. With the above definition of *w*_*ij*_, a cyclic shortest path in the graph *G* corresponds to a continuous and smooth dark contour around the seeding point *p* in the original image (see Fig. 5).

To ensure that the sought contour for the seeding point *p* is closed, we need to make sure that the starting point and ending point of the shortest path computed in *G* is at the same location. This can be done by augmenting the graph *G*, as follows. We add one more column to *G* as the (*n*+1)-th column, which is merely a replica of column 1, and add edges from column *n* to column *n*+1 in the same way as specified above. Then, the formal problem definition is: In the (augmented) graph *G*, find the path with the smallest length among all the shortest *V*_*i*,1_-to- *V*_*i*,*n*+1_ paths in *G*, for all *i*=1,2,…,*m*. Note that *G* has *n*+1 columns, every column has *m* nodes, and each node has *O*(1) outgoing edges. A naive approach would be to compute a shortest *V*_*i*,1_-to- *V*_*i*,*n*+1_ path for every *i*=1,2,…,*m*, and then find the path with the smallest length among these *m* shortest paths. Since the multi-column graph *G* is a directed acyclic graph, computing each shortest *V*_*i*,1_-to- *V*_*i*,*n*+1_ path takes *O*(*mn*) time. Thus, overall this naive approach runs in *O*(*m*^2^*n*) time.

The above algorithm can be improved. A key observation is that the “non-crossing” lemma for the shortest paths in the multi-column graph in [5] also holds for the graph *G* here, because the multi-column graph in [5] and *G* are similar in their structures. Hence, we are able to reduce the time bound of the algorithm for computing an optimal closed contour to *O*(*mn* log*m*), as follows. We first obtain the shortest *V*_*m*/2,1_-to- *V*_*m*/2,*n*+1_ path, and use this path to partition the graph *G* into two subgraphs *G*_1_ and *G*_2_; then we compute the shortest $V_{i_{1},1}$-to-$V_{i_{1},n+1}$ paths for all *i*_1_<*m*/2 in *G*_1_ and the shortest $V_{i_{2},1}$-to-$V_{i_{2},n+1}$ paths for all *i*_2_>*m*/2 in *G*_2_, recursively. In this divide and conquer scheme, a graph is partitioned into smaller subgraphs at each dividing step. It is easy to see that for each recursion level, the algorithm takes in total *O*(*mn*) time, and there are *O*(log*m*) recursion levels. Thus, the overall time bound of this improved algorithm is *O*(*mn* log*m*).

Therefore, the above local search process obtains an optimal path corresponding to an optimal closed smooth contour in the original image which contains a seeding point *p* and has the minimum total cost among all possible feasible contours for *p*. For every seeding point *p*_*i*_, we record the curve for its optimal closed contour as *Boundary*_*i*_ or *B*_*i*_ and the enclosed region as *Region*_*i*_ or *R*_*i*_. Suppose *K* seeds are sampled. Then we have *B*_*i*_ and *R*_*i*_, *i*=1,2,…,*K*. How well these boundaries and regions capture the true glands in the input image is determined by many factors, e.g., the quality of the original image, seeding positions, cost functions used, the value of the smoothness constraint parameter, etc. It is possible that not all these resulted boundaries are correct gland boundaries. In the next section, we develop an ensemble procedure to collect and collaborate these boundaries and their enclosed regions, in order to build a more stable and useful gland boundary probability map (BPM).

#### Ensemble

The basic idea of the ensemble method is: When there are different sources from different perspectives to give suggestions on one object, e.g., a point (pixel) is on a true gland border or not, the ensemble process collects these suggestions and forms a more unbiased and more accurate conclusion [7].

In our situation, the local search for an individual seeding point *p* can be viewed as a maker of suggestions. The search operates on the local image domain around *p* with a radius *r*, and gives suggestions on certain points (pixels) in this domain on whether they are on a gland border contour. The points on the contour curve obtained by the search are assigned a value of 1 (for “yes”), and the points enclosed by the closed contour but are not on its boundary are assigned 0 (for “no”). Suppose a point (pixel) *p*_*i*_ of the input image is covered by *k* such contour-enclosed regions computed by all the local searches. Then *p*_*i*_ receives *k* such suggestions. Overall, the probability of *p*_*i*_ being on a true gland border contour is defined as:

4 $$ P(p_{i} \: on \: true \: gland \: boundary)=(\sum\limits_{k'=1}^{k} f(p_{i},k'))/k  $$

where

$$f(p_{i},k') = \left\{ \begin{array}{l l} 1 & \quad{p_{i} \in B_{k'}}\\ 0 & \quad{p_{i} \in R_{k'}} \end{array} \right.$$

$B_{k'}\phantom {\dot {i}\!}$ denotes the $k' th\phantom {\dot {i}\!}$ searched closed contour; $R_{k'}\phantom {\dot {i}\!}$ denotes the enclosed region inside of $B_{k'}\phantom {\dot {i}\!}$ (not including $B_{k'}\phantom {\dot {i}\!}$). We perform the above processing for every point (pixel) in the image, and obtain a boundary probability map *BPM* for the entire image.

#### Producing segmentation proposals

**Fig. 7:**
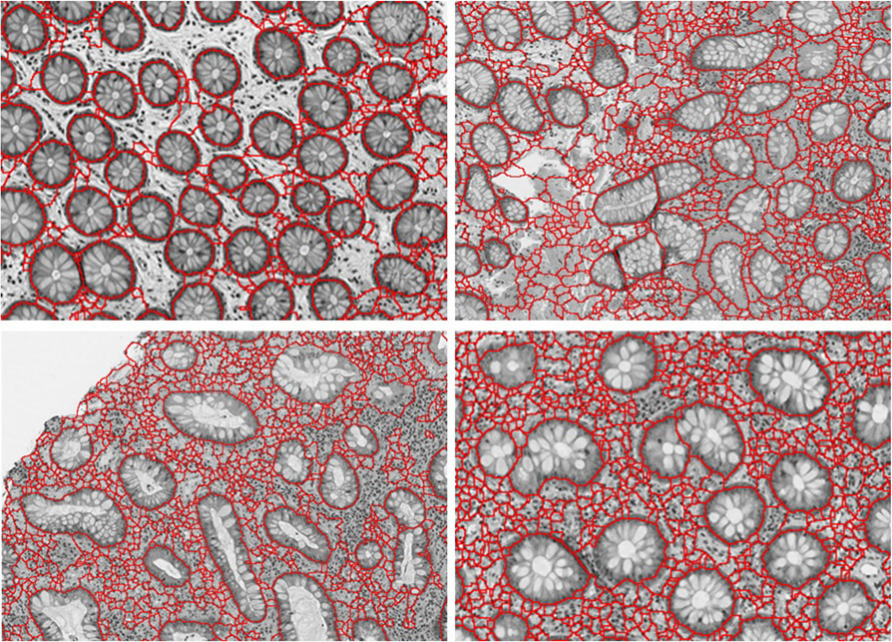
Illustration of gland segmentation proposals: Each area in the image examples enclosed by a red contour is a segmentation proposal region (whose type as gland or non-gland will be determined later)

Based on the gland boundary probability map *BPM*, we next generate segmentation proposals. It was shown in [1] that with a good boundary map, segmentation proposals can be generated effectively and reliably. We adopt the original watershed method [14] to generate segmentation proposals using the gland boundary information. In the boundary probability map, we set all values which are smaller than a threshold *T* to be 0. In practice, we set this threshold *T* simply as 0.5. This basically means that pixels with their probability values smaller than 0.5 are less likely to be on any gland border. Due to the nature of the watershed method, we set all values below 0.5 to be 0 in order to avoid too many meaningless gland regions to be generated (i.e., over-segmentation). We then apply the regular watershed method to the truncated boundary map thus resulted to obtain non-overlapped segmentation proposal regions. Figure 7 gives image examples to illustrate the ability of our method on generating good boundary-preserving gland segmentation proposals. One may see that our segmentation proposals can cover many weak boundaries of glands.

**Fig. 8:**
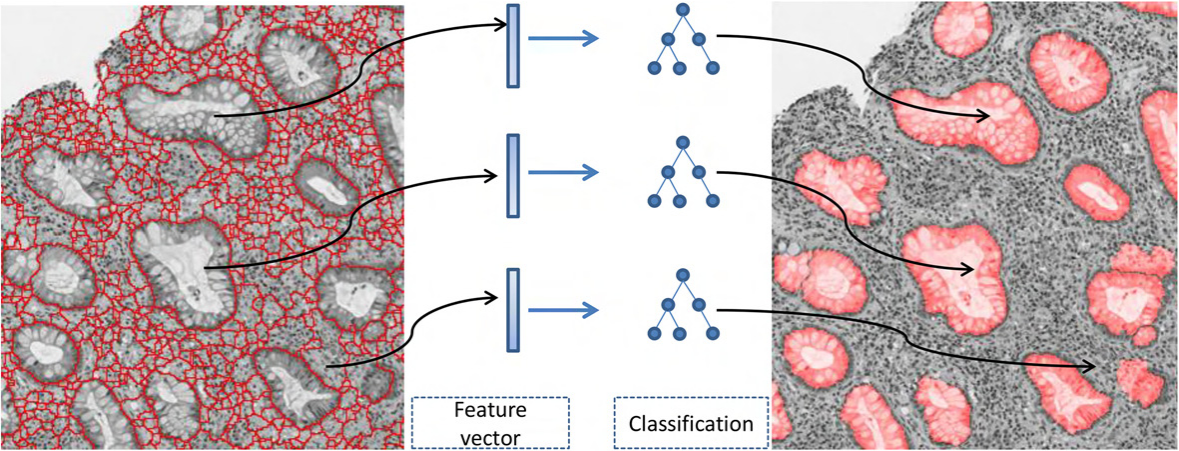
After generating segmentation proposals (*left*), feature extraction and classification processes are conducted to determine the actual gland locations in the image (*right*)

In this step, we have segmentation proposals generated. The next step will determine the type of every segmentation proposal region as gland or non-gland (see Fig. 8).

### Feature extraction and classification

This step determines each segmentation proposal region as gland or non-gland. This task falls into the category of two-class classification problems. For such problems, a common approach is to first extract some features from the original image region under consideration, and then determine its type based on a training/testing classification procedure in the feature space. We also adopt this approach. Yet for a more effective and fast processing, we first prune away a significant portion of the proposals which are very unlikely to be part of any glands, and then perform feature extraction and classification on the remaining proposals.

#### Proposal pruning

This step aims to eliminate a significant portion of candidate segmentation proposals which are very unlikely to belong to any true gland based on some simple and fast criteria, in order to speed up the whole process. As shown in Fig. 7, many non-gland segmentation proposal regions are very small; thus we can safely prune away segmentation regions of small sizes. Also, using the boundary probability map *BPM*, we can obtain the average probability value for the points on the boundary of each proposal region. Usually, such an average value for a gland proposal region should be large, but for a non-gland region in general should be much smaller. Specifically, we take a proposal region as non-gland if its such average value is smaller than 0.3. Our experiment showed that this simple pruning step can successfully remove at least half of the candidate proposals safely.

#### Feature extraction

To classify the remaining “harder” cases, more advanced techniques and criteria are employed. On each such segmentation proposal region, we extract a set of visual features for determining its region type (as gland or non-gland). In general, we consider two types of features for this.

1. Basic in-region and on-boundary information.

2. Advanced image descriptors.

##### Basic in-region and on-boundary information.

Lumen usually appears inside a gland, and it looks bright in the image (high intensity). The cytoplasm of an epithelial cell is also bright due to H&E staining (see Fig. 2). Thus, as a whole, a gland region may have a brighter mean intensity than the background. Also, due to the nature of our segmentation method, the sizes of true gland proposals are usually larger than those of the regions from the background. That is, the sizes of proposal regions are also a useful feature. A gland normally has a continuous dark cell nucleus contour border. To capture this property, we compute the mean intensity of the border contour for each proposal region, as well as the corresponding standard deviation. In all, we extract features as the mean intensity of each proposal region and its standard deviation, the size of the region, and the mean intensity of its boundary and the standard deviation.

##### Advanced image descriptors.

**Fig. 9:**
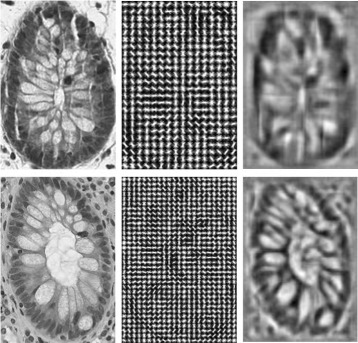
Illustration of the HOG feature. *Left*: original images; *middle*: HOG features extracted from the original images; *right*: visualization (inverse) of the HOG features [18]. Observe that the HOG feature can represent the gland structure quite well

Advanced image descriptors can present texture and more detailed information than the basic ones above. HOG (histogram of oriented gradients) [6] is one of the most widely used image descriptors. PHOG (pyramid histogram of oriented gradients) [2] was used in [9, 10]. We choose to use the original (simpler) version of the HOG feature (see Fig. 9). Recently, learning based features, especially deep-learning features, have been studied to represent more complex appearances such as general objects and human faces (e.g., identity or expression). We think the appearances of glands are relatively simple and stable, and thus simple HOG feature is good enough to represent such structural information. The HOG feature can be in high dimensions; of course, not every dimension of the feature space is equally important. PCA (Principal component analysis) may be applied to the HOG feature space to reduce the feature dimensions and make the feature more efficient and effective to use.

Overall, we stack these two types of features into a long vector, which is input to our classification model. The classification model receives the feature vector for each proposal region, and predicts its type (gland or non-gland).

#### Classification

We now discuss how to assign a type label to each proposal region using its feature vector. Suppose there are *M* gland regions in the ground truth set *GT*, and *R*_*gt*_∈*GT* is a region area which covers exactly one gland in the original image. For a proposal region *R*_*proposal*_, we define the score *P*(*R*) for this proposal region as:

5 $$ \max_{R_{gt} \in GT} \frac{R_{proposal} \cap R_{gt}}{R_{proposal} \cup R_{gt}}  $$

A proposal region with a larger *P*(*R*) score means it is more likely to be a gland region. We need to learn a classification model to predict the *P*(*R*) value based on the extracted feature of the proposal region *R*_*proposal*_.

Multiple classification models can be considered for this purpose, e.g., Logistic Regression [12], SVM [4], Random Forest [3], etc. We choose Random Forest as our classification model because it is fast, stable, and invariant to the value ranges of the feature space. Since the *y* value is in the range of 0 to 1, we use the regression version of Random Forest.

### Refinement

The regression model provides a score for every proposal region. Since the score is assigned independently to each proposal region, we use a post-processing to collaborate the results of neighboring regions and improve the gland detection accuracy (eliminating potential false positives).

Usually, artifacts (false positives) may occur near some true gland nucleus borders. This is mainly due to the fact that gland borders are an important sign for the existence of glands, and this sign can also make some areas that are near true glands but are not true glands be classified as gland (false positives).

**Fig. 10:**
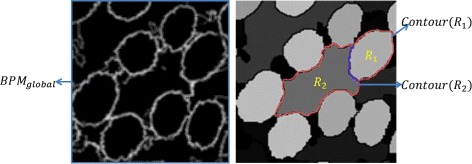
The input to the refinement step. For two neighboring proposal regions, their outer contours may overlap (e.g., the purple curve in the right image). The re-weighting process uses such overlap and the map *BPM* to re-weight the gland probability for each proposal region



If we view gland borders as a “resource” associated with image locations, then once a true gland region “consumes” this resource, a nearby artifact region could become lack of gland borders (i.e., no gland border is available for forming its closed boundary as a gland). Based on this observation, we propose a re-weighting procedure to re-weight the region probability map generated by the regression. The purpose of the re-weighting process is to further highlight true gland regions and weaken artifact ones. We use the boundary probability map *BPM* again here to help the re-weighting. In Fig. 10, the gland border resource is represented by *BPM*, and an artifact region *R* is surrounded by 6 true gland regions. In the order of the scores assigned by the regression model (from high to low), the 6 true gland regions can take the gland border resource away from the outer contour of *R*_2_ (the red contour in the right image of Fig. 10) before *R*_2_ seeking the gland border resource; when it is *R*_2_’s turn, there is almost no gland border to use. Based on this mechanism, we design a simple algorithm for the gland probability re-weighting process, as follows.

**Fig. 11:**
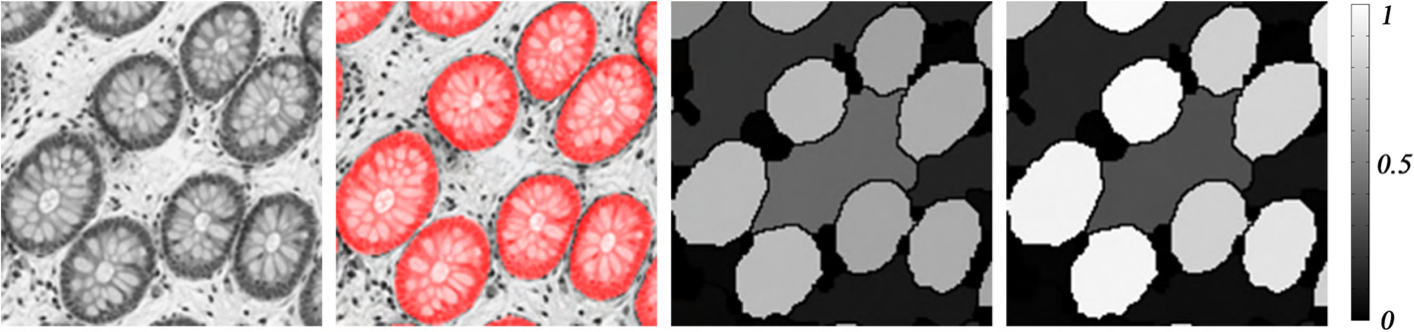
Illustration of the refinement. Images from left to right: an original intensity image, ground truth (*in red*), the gland probability of segmentation proposals before re-weighting, and the gland probability of segmentation proposals after re-weighting, respectively. The intensity bar represents the probability of gland detection, with *black* = low probability, *gray* = intermediate, and *white* = high probability of gland detection

Figure 11 gives examples of the effects after applying the re-weighting process. Initially, an artifact region surrounded by true gland regions may have just a slightly lower probability value comparing to the values of these gland regions. But after re-weighting, the differences of such values between the artifact region and gland regions become larger (controlled by the decreasing parameter *λ*_*d*_).

### Merging proposal regions

**Fig. 12:**
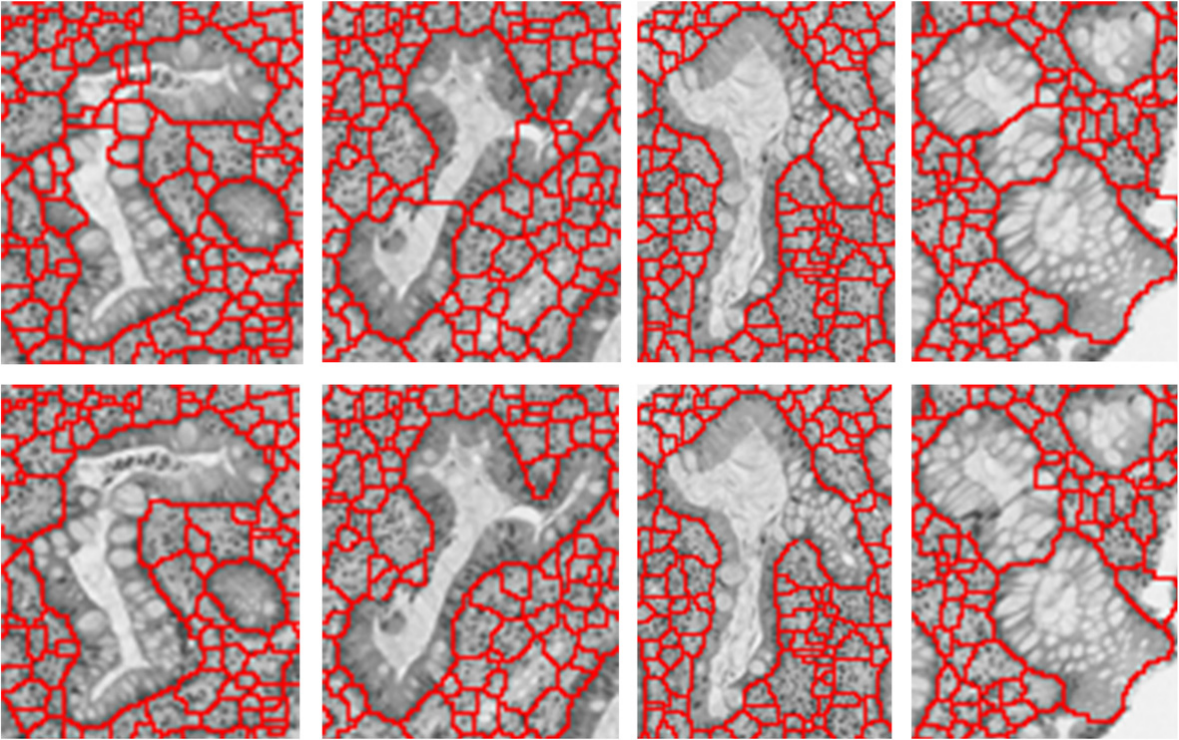
*Top row*: proposal regions before the merging step; *bottom row*: proposal regions after the merging step

A potential problem of our seeding-searching-ensemble method is that it might generate over-segmented proposal regions for irregular-shaped glands (e.g., see Fig. 12). Although such irregular-shaped glands do not appear quite often, they are strong indications for chronic inflammatory diseases (e.g., inflammatory bowel disease). Thus, it is necessary to merge over-segmented gland regions.

Given a set of proposal regions *R*_*i*_, *i*=1,2,…,*m*, we apply a greedy merging method to merge potential over-segmented proposal regions. We say that two regions *R*_*p*_ and *R*_*q*_ are neighbors if their contours *C*(*R*_*p*_)∩*C*(*R*_*q*_)≠*∅*. Each pair of neighboring regions has a score *P*_*pq*_ indicating how likely these two regions belong to the same gland (and thus should be merged). For simplicity, we compute *P*_*pq*_ based on the boundary probability map (more details of this are given below). In every iteration, the pair of neighboring proposal regions *R*_*p*_ and *R*_*q*_ with the highest score *P*_*pq*_ is merged, and then the relations of the merged region thus obtained to its adjacent regions are updated. We repeat this process until the highest *P*_*pq*_ is smaller than 0.5. We present the algorithm for this merging method, as follows.



Figure 12 shows several examples of over-segmented regions for irregular-shaped glands merged by our merging method.

### Results and Discussion

We collected 10 clinical H&E-stained histology images of human intestinal tissues digitized at 40x magnification with a medical grade slide scanner (Aperio, Leica Microsystems Inc. Buffalo Grove, IL). At 40x magnification, 1 mm = 4050 pixels. This data set consisted of images with normal colon (4 patients), colon with histological features of chronic inflammation (3), colon with acute inflammation (2), and ileum with acute inflammation (1). In total, there are 1723 glands. Human experts manually labeled these glands to form the ground truth. The variations of gland appearances and the environment of the tissue sections are quite large across the images, and provide a robust data set to test our methods. This is due the following reasons: (1) The images were sampled from regions of the gut including colon and ileum; (2) Inherent in the biopsy and tissue processing techniques is that tissue is sampled in any orientation which results in display of tissue in any plane through the original tissue. (3) The samples reflect a spectrum of normal and disease states as they were obtained from a patients who were deemed to have normal gut or gut affected by a range of acute to chronic inflammation.

This project was reviewed and approved by the Institutional Review Board and compliant with the privacy provisions of the Health Insurance Portability and Accountability Act (HIPAA) of 1996. Tissue specimens were obtained under an IRB-approved waiver of consent applicable to de-identified samples.

### Settings

Since our gland proposal generation does not require any training phase, we apply the same setting to all images for the gland proposal generation process. The radius *r* for the local search is set as 100 pixels, because the long diameter of a gland in our images is between 80 to 160 pixels. The number of sampling rays is set to be 360, that is, every ray covers 1 degree in the polar plane. The parameter *λ* in the cost function is set as 10. Before applying the watershed method to generate the segmentation, the threshold value on the boundary probability map is set to be 0.5. The pruning threshold on the gland segmentation proposals is set as 0.3. For the final refinement, the decreasing parameter *λ*_*d*_ is set to be 1/2. Most of the parameters are intuitive and easy to set.

For the classification process, the random forest needs to be trained using data. We adopt the common 10 fold cross-validation. That is, for each time of the testing, we use 9 images as training data, and the remaining 1 image as testing. We repeat this training and testing process ten times, each with different testing images and the corresponding different set of training images. Below we report the gland detection performance and gland segmentation performance of two state-of-the-art methods and our method.

### Gland detection

We adopt the PASCAL VOC criteria [8] for evaluating the gland detection performance, which is commonly used in object detection studies and were also adopted in [10]. A detected region is viewed as true positive if $\frac {R_{detected} \cap R_{gt}}{R_{detected} \cup R_{gt}} >0.5$.

**Fig. 13:**
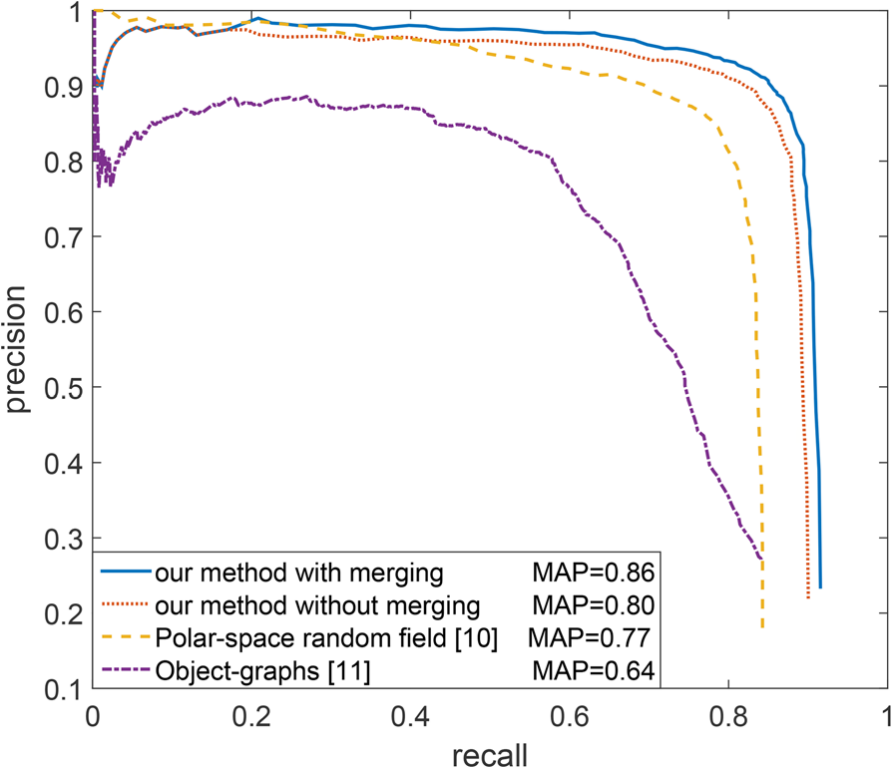
Precision-recall curves for gland detection

The precision is calculated by *TP*/(*TP*+*FP*), and the recall is calculated by *TP*/(*TP*+*FN*). Figure 13 and Table 1 show the detection performance of our method comparing to [10] and [11]. When determining a region is gland or non-gland, the method proposed in [11] used only 8 simple features rather than more advanced image descriptors. This is one reason why its detection performance is far lower than the other two methods. More advanced image descriptors such as HOG (histogram of oriented gradients) can effectively represent more complicated structural information, and are widely used in many computer vision tasks. Comparing our method to the method in [10], both methods utilize HOG feature in the classification procedure, but our method performs more stably and has a higher mean average precision (MAP) by 9 % (see Fig. 13), a higher recall by 6 %, and a higher precision by 6 % (see Table 1). We think this advantage is due to our better segmentation process.

**Table 1:** Detection performance comparison

	Recall	Precision	F-score	
OG [11]	0.662	0.690	0.676	
PSRF [10]	0.787	0.845	0.815	
Our method without merging	0.835	0.892	0.862	
Our method with merging	0.850	0.908	0.878	

### Gland segmentation

**Fig. 14:**
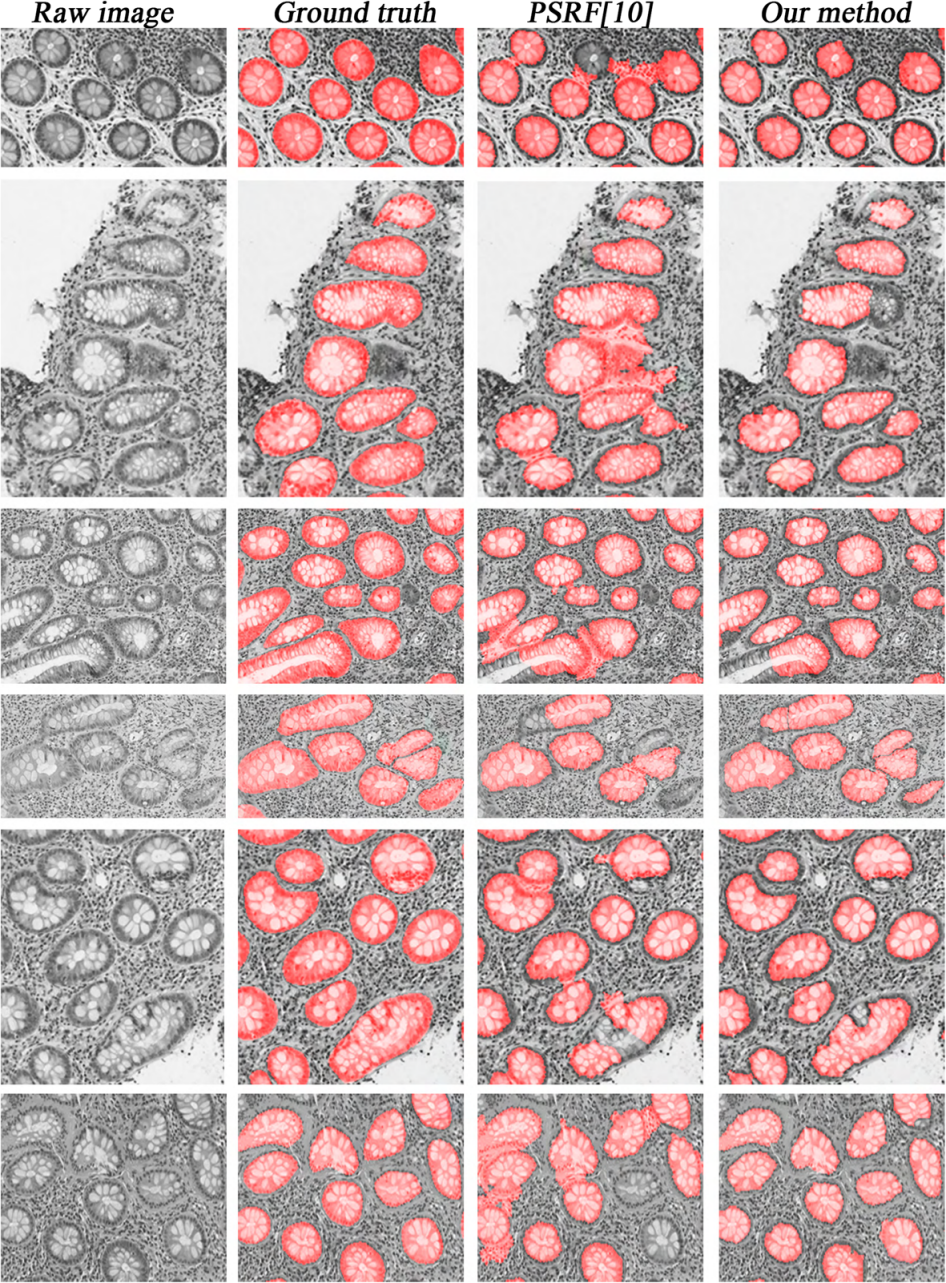
Six examples which compare the results of two segmentation methods. The columns from left to right: original intensity images, ground truth (*in red*), results by PSRF [10], and results by our method, respectively. Our new method has less leaking and preserves more gland boundaries

The segmentation performance is measured at the pixel level, and is concentrated more on examining gland boundary preservation (to prevent leaking). We compute the true positives, true negatives, false positives, and false negatives using pixel-by-pixel comparison of the algorithms’ results against the ground truth. Table 2 shows the segmentation comparison result when the three methods achieve their best F-scores in detection. One can see that our method obtains more accurate gland segmentation. Figure 14 shows some segmentation visual comparison examples. Our method is significantly better at preserving true gland boundaries and preventing segmentation leaking than [10].

**Table 2:** Segmentation performance comparison

	Recall	Precision	F-score	
OG [11]	0.652	0.662	0.657	
PSRF [10]	0.776	0.794	0.785	
Our method without merging	0.826	0.853	0.840	
Our method with merging	0.838	0.874	0.856	

## Conclusions

In this paper, we proposed a new seeding-searching-ensemble method for gland segmentation and detection in H&E-stained histology images. We showed that on generating gland segmentation, local search with a hard shape constraint followed by an ensemble procedure is more robust on preserving gland boundaries and preventing leaking. We produced high-quality segmentation and detection of non-overlapped glands that obey the natural property of glands in histology tissue images. With accurately detected and segmented glands, quantitative measurement and analysis can be developed for further studies of glands and computer-aided diagnosis.
